# Dementia Precox

**Published:** 1916-09

**Authors:** F. S. White

**Affiliations:** Late Superintendent of the Southwestern Insane Asylum, San Antonio, Texas


### Dementia Precox.

BY DR. F. S. WHITE, LATE SUPERINTENDENT OF THE SOUTHWESTERN INSANE ASYLUM, SAN ANTONIO, TEXAS.

"Dementia precox is a disease beginning usually early in life and characterized chiefly by a more or less marked and peculiar enfeeblement of the mind, but manifesting upon this basis a considerable variety of symptoms, such as emotional indifferences, weakness of judgment, flightiness, verbigeration, automatic obedience, catalepsy, echopraxis, stereotypy, negativism, mutism, impulsive actions, affectations, grimaces and unemotional laughter, delusions of a depressed or grandiose nature and hallucinations."

A disease which attacks and disables especially the young and growing manhood and womanhood of the country and which accounts for at least 25 per cent of the admissions into the insane asylums demands a most careful and scientific study and investigation. The psychosis is divided into three main groups: Hebephrenic, katonic and paranoidal. This division is more a matter of convenience in classification, as the types are not clear and distinct but shade imperceptibly one in the other and all tend towards progressive mental deterioration. The dementia is of a peculiar type, as there is an absence of actual quantitative loss of mental function. Many and various theories have been advanced as to the cause of dementia precox. All of them have some merit, but so far none of them have been entirely satisfactory and conclusive. Kraepelin contends that we have to deal with an actual chemical injury to the cortical cells, causing their deterioration or destruction and that the origin and development of the psychosis are best explained by the theory of an autointoxication, arising possibly in connection with processes going on in the sexual organs. He is led to the last idea by the unusually close association of the disease with the age of development, with menstrual disorders and with pregnancy and childbirth.

A theory advanced from the Freud psychology is that many cases are of psychogenic origin; that is, that they owe their origin to repressed emotional complexes, and to account for the actual deterioration he conceives that same toxin is ultimately created in the emotional condition. There is no doubt some truth in all these theories, and they should be given due consideration in an attempt to arrive at the true cause of the psychosis.

My opinion, based upon the study of a large number of cases and extending over a period of many years, is that the basic cause of dementia precox is found in the hereditary condition of the individual; the unstable, nervous organization inherited from the ancestry.

The other factors mentioned are only exciting causes and without the bad heredity would not produce the disease. This opinion is borne out by the fact that practically every case of dementia precox has in earlier life shown some peculiarity; some departure from the normal. By careful and painstaking investigation it will be ascertained that, as children, these cases were peculiar in some respects. As a rule, they are over-sensitive and take the ordinary affairs of life too seriously. They think that they are discriminated against by their parents, or other members of the family. They have the idea that their playmates and associates do not treat them right; that they are imposed upon and make mountains out of the most trivial occurrences of life. At the age of puberty, which is one of the critical stages of life in every individual, these hereditary tendencies are more apt to develop and assume very important proportions. They become introspective and self-centered and develop a condition which has been described as a "shut-in personality."

August Hoch has well expressed this idea: "We find," he says, "in dementia precox, persons who have no natural tendency to be open and get into contact with the environment, who are reticent and seclusive and cannot adapt themselves to situations; who are hard to influence and often sensitive and stubborn, but the latter more in a passive than in an active way. They show little interest in what goes on and frequently do not participate in the pleasures, cares and pursuits about them; although often sensitive, they do not let others know what their conflicts are; they do not unburden their minds, are shy and have a tendency to live in a world of fancies."

These cases are found sitting in the wards of hospitals for the insane for hours without once moving or changing position. They seem to be wrapped in the solitude of their own thoughts and are absolutely oblivious of what goes on about them. Emotional disturbances are not uncommon and often certain cases will suddenly break out in the most violent fits of laughter. They may also have spells of the most heartrending crying and exhibit every indication of distress. Also they may have sudden outbreaks of anger, and on such occasions may suddenly strike anyone near them. After these various emotional outbreaks they lapse again into apparent mental oblivion. The cause of these outbreaks are concealed, and attempts to get an expression from them is regarded as impertinence or meddling, which is bitterly resented.

In many instances these outbreaks are the result of delusions or hallucinations. In others they are the result of certain lines of thought or recollections of instances in their past life. A very marked and prominent symptom of this psychosis is negativism; that is opposition to anything and everything. They resist going to their meals, for instance, and they will just as actively resist leaving the dining room. They will resist the removal of their clothing at night and resist with all their strength putting it on in the morning. This opposition and resistance extends to every act during the day.

Another prominent symptom in many cases is stereotypy, in which condition "the automatic impulse will be reiterated over and over again in either language or conduct." There is no means by which it can be determined with any degree, even of probability, what a case of dementia precox may do. "It is in dementia precox that the extraordinary happens. The sudden motor explosions, impulsive action, irrelevant laughter, stereotypy of speech and movements, negativism, catalepsy, echolalia, echopraxia, strange imperative ideas, the symptoms of thought deprivation, and the salutary fancies, affectations, mannerisms, and so on. Now doubtless all of these curious phenomenas have their psychological explanation if we could only get at them."

The prognosis in dementia precox is bad; so much so that some alienists contend that recovery is extremely rare. A certain per cent of cases do recover. A larger per cent improve, and some have remission of longer or shorter duration.

In the treatment of this disease, prophylaxis plays a very important part. As we cannot control the transmission of hereditary defects to offspring, we must take these cases as we find them and do the best we can to restore them to their normal status. The development of dementia precox can be prevented in many cases where the tendency exists, by proper advice and care in early childhood. Many parents often neglect the peculiarities of the child, as something to be proud of; as something that distinguishes the child from other children. In these peculiarities, however, the competent and painstaking physician sees a real danger signal. After the case is once developed the results are much more doubtful and uncertain. These cases must be taken away from their family and relatives and given such remedies as are indicated to put the general system in good condition. I believe that the extracts of the ductless glands are destined to be of great benefit. Unfortunately, at present our methods of diagnosis are not sufficiently accurate and defined for us to know just what extract is indicated in individual cases. I am certain that I have seen splendid results follow the administration of thyroid extract in some cases, also ovarian extracts do much good in certain cases in young girls and women. These case must have diversion, and every effort should be made to relieve the introspection and enable the thoughts to be directed into different channels. Properly directed occupation and amusements are of great value. In even chronic cases much can be done in the way of what is termed re-education. The will, which often seems to be paralyzed, must be redeveloped. These means require the most patient and persistent efforts. An enormous amount of good nourishment is an absolute necessity. The mental strain and concentration is very exhausting, not only to the physical health, but to the mental strength. No close and fast rules can be laid down for treatment, but each case is a law unto itself, and it will tax the ingenuity of the medical attendant to know what course offers the best probable results.

Medicines have a very important place in the treatment not as directed to the specific disease, but in the way of putting the general health on the upgrade and thus make better blood, which in turn will improve the tone and quantity of the brain cells.

References: August Hoch, Kraepelin, Frederick Peterson.

